# Malaria: modification of the red blood cell and consequences in the human host

**DOI:** 10.1111/j.1365-2141.2011.08755.x

**Published:** 2011-05-28

**Authors:** Christopher A Moxon, George E Grau, Alister G Craig

**Affiliations:** 1 Malawi-Liverpool-Wellcome Trust Clinical Research Programme, College of MedicineChichiri, Blantyre 3, Malawi; 2 Liverpool School of Tropical MedicineLiverpool, UK; 3 Vascular Immunology Unit, Department of Pathology, Bosch Institute, Sydney Medical School, The University of SydneySydney, NSW, Australia

**Keywords:** *P. falciparum*, PfEMP1, sequestration, cerebral malaria, RBC polymorphisms

## Abstract

Residence in the human erythrocyte is essential for the lifecycle of all Plasmodium that infect man. It is also the phase of the life cycle that causes disease. Although the red blood cell (RBC) is a highly specialized cell for its function of carrying oxygen to and carbon dioxide away from tissues, it is devoid of organelles and lacks any cellular machinery to synthesize new protein. Therefore in order to be able to survive and multiply within the RBC membrane the parasite needs to make many modifications to the infected RBC (iRBC). *Plasmodium falciparum* (*P. falciparum*) also expresses parasite-derived proteins on the surface of the iRBC that enable the parasite to cytoadhere to endothelial and other intravascular cells. These RBC modifications are at the root of malaria pathogenesis and, in this ancient disease of man, have formed the epicentre of a genetic ‘battle’ between parasite and host. This review discusses some of the critical modifications of the RBC by the parasite and some of the consequences of these adaptations on disease in the human host, with an emphasis on advances in understanding of the pathogenesis of severe and cerebral malaria (CM) from recent research.

For much of its life in the human host *Plasmodium falciparum* is intracellular, with only brief direct exposure of the ‘naked’ parasite to the immune system. Several candidate vaccines have attempted to use these short periods of vulnerability to produce therapies based on surface antigens of the sporozoite and merozoite forms, with varying success. Some of the difficulty with targeting this phase of the life cycle may be explained by recent work on the proteolytic modification and mobilization of merozoite proteins (Riglar *et al*, 2011), which showed how the parasite displays the mature active form of the protein on its surface only at the time when its function is required. Therefore it is curious that during the erythrocytic cycle *P. falciparum* modifies the red blood cell (RBC) (Fig. 1) through the insertion of parasite-derived proteins into and onto the membrane. This latter property has led to the development of a sophisticated system of antigenic variation (for review see (Dzikowski & Deitsch, 2009), with programmed switching of surface antigens that minimizes recognition by the immune system and allows for chronic infection of the host. That an intracellular parasite displays external antigens that are visible to the immune system is intriguing and may reflect the need to modify the environment in the highly differentiated human erythrocyte and the highly variable state of the circulation. Parasite modifications both enable the parasite to modify the intra erythrocytic environment from one specialized to hold haemoglobin and assist in gaseous exchange to one in which the parasite can synthesize it’s progeny; but also to stick in the peripheral microcirculation where it minimizes shear forces, has a constant high carbon dioxide and low oxygen environment optimum for growth and avoids the splenic filter which would remove and destroy the infected RBC (iRBC).

**Fig 1:**
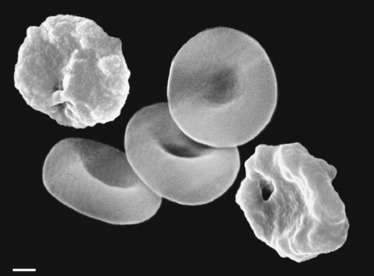
Scanning electron micrograph of normal RBC and *Plasmodium falciparum* iRBC. The three normal RBC at the centre appear regular and smooth and have a biconcave structure. In contrast the two peripheral iRBC have an irregular and rough surface and have lost the biconcave structure. (Published with permission, Professor David Ferguson, Oxford University, Oxford, UK). Scale bar = 1 μm.

## Modification of the red cell surface: PfEMP1 and other surface proteins

The presence of antigenically diverse proteins on the surface of the iRBC and its implications for antigenic variation were first alluded to by Brown and Brown (1965) in the primate malaria *P. knowlesi*. This largely biochemical and immunological characterization was translated to *P. falciparum* in the mid-1980s (Leech *et al*, 1984) with the first biochemical description of *Plasmodium falciparum* erythrocyte membrane protein 1 (PfEMP1). Molecular characterization of PfEMP1 took another 10 years and was greatly facilitated by early efforts at genome sequencing, which identified a number of large, variable open reading frames with characteristics that matched PfEMP1 (Baruch *et al*, 1995; Smith *et al*, 1995; Su *et al*, 1995), namely a large and diverse external segment composed of different domains (termed Duffy binding-like (DBL) with sequence similarity to regions found in proteins known to mediate adhesive interactions during invasion. This link with adhesion was particularly important and subsequent work has identified the binding sites in PfEMP1 for a number of host receptors involved in endothelial cytoadherence, rosetting (the adherence of iRBC to uninfected RBC) and placental sequestration (Rowe *et al*, 1997; Smith *et al*, 1998, 2000; Salanti *et al*, 2003). The phenomenon of sequestration, first described in the late 19th Century by the Italian pathologists Marchiafava and Bignami (1892), has been associated with pathological features of severe malaria (see below) and the molecular events are largely ascribed to PfEMP1, although it is possible that other parasite and host-derived molecules may participate in this important interaction. Other iRBC proteins include RIFINS, STEVOR, Pfmc-2TM and SURFINS (for review see Templeton, 2009), the functions of which are largely unknown but are encoded by gene families some (e.g. *rifs*) exceeding the repertoire of *var* genes, which encode PfEMP1.

### Pathogenesis: Consequences to the host of parasite surface proteins – cytoadherence and sequestration

#### Endothelial cytoadherence and sequestration

The importance of cytoadherence and sequestration in human *P. falciparum* disease is widely described and since Marchiafava and Bignami’s report in 1892 many pathological series of patients dying from severe malaria have described parasites sequestered in the microvasculature of vital organs (Dudgeon & Clarke, 1917; MacPherson *et al*, 1985; Taylor *et al*, 2004). Sequestration is also apparent because in *P. falciparum* mature forms of iRBC are virtually absent from the peripheral circulation. However, the other Plasmodium species that can cause severe disease in humans, *P. vivax* and *P. knowlesi*, do not cytoadhere to the same extent, with mature forms readily seen in the peripheral circulation, nor do strains that cause murine models of severe malaria. Hence, a long raised question has been whether severe falciparum malaria can occur without sequestration and particularly whether sequestration is required for cerebral malaria (CM); the most serious complication of *P. falciparum* malaria, which causes coma and seizures and has a mortality of approximately 20%. This question has been elusive because the only definitive way to demonstrate sequestration has been by observing parasites in vessels from tissues from patients who have died. It is also difficult because malaria-infected patients may have other pathologies, such as co-infection with bacteria, and it can difficult to separate the effects of the different components. In this light, a significant advance in understanding malaria pathogenesis is the recognition that retinal changes, detected clinically by opthalmoscopic examination, are a sensitive and specific way of detecting sequestration in cerebral vessels (White *et al*, 2001; Beare *et al*, 2006). A large pathogenesis study in Malawi in which retinal examination was performed on malaria-infected children with presumed CM and then autopsies were done on children who went on to die, found that at post mortem there was a very high correlation between cerebral sequestration and retinal changes (Taylor *et al*, 2004). Furthermore, children without retinal changes nearly all had another identifiable cause of death; implying that they in fact had another disease with an incidental parasitaemia and that CM does not occur in humans in the absence of cerebral sequestration.

Additional evidence for the importance of cytoadherence comes from recent studies of human RBC polymorphisms. It has long been recognized that maintenance in the human gene pool of the haemoglobin polymorphism HbS occurs because the heterozygous state HbAS, sickle cell trait, confers protection against severe malaria. However the mechanism for this protection has been controversial. While the impairment to growth of *P. falciparum* parasites in HbAS iRBC in hypoxic conditions provides a partial explanation (Pasvol *et al*, 1977; Friedman, 1978) there has been debate as to whether this can explain the magnitude of protection conferred by sickle cell trait. It also does not explain how protection increases progressively through childhood in individuals with HbAS relative to individuals with HbAA, as demonstrated in a large cohort study in Kilifi Kenya (Williams *et al*, 2005). This acquired protection implies an immunological basis. More recently it has been shown that HbAS *P. falciparum* iRBC have an altered display of PfEMP1 on the iRBC surface and that this interferes with cytoadherence to endothelial cells and blood monocytes (Cholera *et al*, 2008). This failure of HbAS iRBC to cytoadhere is particularly interesting because not only does it explain a decreased likelihood of severe disease in any given infection, by preventing a key pathogenic mechanism, it also forces mature iRBC to pass through the spleen. In this way HbAS individuals might more effectively present antigens from mature parasite forms to the immune system after digestion in the spleen. In support of this concept it has been shown that HbAS individuals have increased levels of antibodies to many parasite antigens (Verra *et al*, 2007). A similar picture of abnormal surface display of PfEMP1 has also been shown for HbAC and HbCC (Fairhurst *et al*, 2005), which also provides protection against malaria in both heterozygous and homozygous states (Modiano *et al*, 2001). That a common mechanism exists for two of the most widespread human genetic RBC polymorphisms highlights the effectiveness of this form of adaptation and implies additional evidence of the critical importance of cytoadherence in pathogenesis.

Recent studies on the RBC polymorphism have also assisted in our understanding of the preferential cytoadherence of iRBC to particular host receptors. The possibility that adherence to a particular receptor may be important in malaria pathogenesis is suggested by: (i) the discovery that placental malaria is caused by presentation of a specific *var* gene –*var2csa*– that confers binding to chondroitin sulphate A, which is expressed at high levels in the syncitiotrophoblast of the placenta (Salanti *et al*, 2004); (ii) the recognition that a parasite normally only expresses a single *var* gene (Voss *et al*, 2006) (iii) the demonstration that iRBC in the peripheral blood of patients with CM tend to have a dominant parasite genotype (Kyriacou *et al*, 2006). However because there are many receptors that PfEMP1 can bind to it has been unclear which binding preference might be implicated in severe disease, particularly CM. Ovalocytosis, a red cell disorder common in South East Asia, has previously been shown to confer protection against CM by selecting for parasites that bind CD36 (Cortes *et al*, 2005). This coincides with studies that have shown preferential binding of parasites from patients with CM to inter-cellular adhesion molecule 1 (ICAM-1) and from patients with uncomplicated malaria to CD36 (Newbold *et al*, 1997; Ochola *et al*, 2011). The implication is that binding to ICAM-1 is associated with CM. As brain endothelial cells express ICAM-1 but not CD36 this might seem obvious, although further work has suggested that platelets may be able to provide CD36 in a cerebral context (see below). It would lead us to expect that polymorphisms that decrease iRBC binding to ICAM-1 would confer protection against CM. Paradoxically, individuals with one such polymorphism, ICAM-1^Kilifi^, which results in the production of an endothelial ICAM-1 molecule with lower affinity to iRBC binding, have an increased risk of developing CM (Fernandez-Reyes *et al*, 1997). Subsequent studies have shown that this allele, found up to 30% of African populations, may be important in protecting against nonmalarial febrile illness in infants (Jenkins *et al*, 2005), highlighting the problem of considering genetic associations with clinical outcomes in isolation.

#### Alternative forms of adhesion

In addition to endothelial cytoadhesion, iRBC can also increase sequestration by binding to or altering circulating host cells. The most notable example of this is the binding of iRBC to CD36 receptors on platelets that have been activated by either thrombin or tumour necrosis factor (TNF) (Lou *et al*, 1997). Scanning electron microscopy has demonstrated that iRBC can utilize this interaction as an alternative method of sticking to endothelial cells, with platelets acting as a bridge to bind iRBC to endothelial cells. In this way CD36-specific iRBCs are able to bind to human cerebral endothelial cells that are CD36-deficient; re-orientating the phenotype of iRBC cytoadhesion and sequestration (Wassmer *et al*, 2004, 2005, 2006).

Platelets also are involved in another mechanism that contributes to sequestration and is thought to be important in malaria pathogenesis; platelet iRBC clumping, in which activated platelets and iRBC bind, forming a large cluster of cells that is unable to pass through narrow microvessels and so becomes sequestered. A captivating link between sequestration and platelet clumping has been proposed by Biswas *et al* (2007) who showed that iRBC can use the ubiquitously expressed C1q receptor – gC1qR/HABP1/p32 – for two crucial elements: cytoadhesion to endothelial cells and platelet-mediated clumping. Its involvement in platelet clumping is particularly significant because previously the only receptor shown to be involved in clumping was CD36. Because both of these processes are related to CM pathogenesis, it is possible that binding to C1qR may be associated with severe disease. The relevance of this mechanism to human disease needs to be further assessed under flow conditions and to be assessed *in vivo*. Conceptually this molecule has the potential for dual binding and for bridging iRBC to endothelial cells by platelets, as implicated in the report by Wassmer *et al* (2004).

Another interesting link by which iRBC can bind to endothelial cells is endothelial membrane fractalkine/CX3CL1 (FKN) (Hatabu *et al*, 2003); immunohistochemistry showed that FKN was expressed in the brain of one patient who died with CM. *In vitro*, more than 20 malaria patient isolates showed binding to FKN-transfected CHO cells, and only mature forms of the parasite were able to bind.

Another example of the potential for platelet-mediated cytoadherence has been reported through the activation of Weibel-Palade bodies in endothelial cells and the release of ultra-large von Willebrand Factor multimers (ulVWF). These ‘strings’ of ulVWF unfold under flow conditions and provide a highly adhesive platform for platelets and iRBC binding, via CD36 (Bridges *et al*, 2010). The activity of the enzyme responsible for the degradation of ulVWF, ADAMTS13, has been shown to be reduced in severe malaria (Larkin *et al*, 2009; de Mast *et al*, 2009; Lowenberg *et al*, 2010). Further work will be required to understand the role of platelet-mediated cytoadherence in disease severity *in vivo*.

## Modification to the red cell membrane and cytoplasm

Figure 1 shows the extent of the remodelling of the infected cell, seen as the presence of novel structures, such as knobs on the erythrocyte membrane, as well as increased rigidity. These knobs are formed through interactions between host and parasite proteins in a complex network involving membrane proteins from the RBC, such as spectrin and actin, and the parasite-derived molecules ring-infected erythrocyte surface antigen (RESA), knob associated histine-rich protein (KAHRP), mature parasite-infected erythrocyte surface antigen (MESA) and PfEMP3 (for review, see Maier *et al*, 2009). Targeted disruption of the genes for these parasite proteins invariably leads to changes in membrane rigidity and, in some cases, cytoadherence. For example, deletion of KAHRP does not block the transport of PfEMP1 to the surface of the infected erythrocyte, unlike PfEMP3 truncation, although this protein is also not required for PfEMP1 trafficking (Waterkeyn *et al*, 2000), and initially was not thought to have a significant effect on adhesion until these assays were carried out under flow conditions when a major reduction was observed (Crabb *et al*, 1997). KAHRP is thought to interact with the cytoplasmic domain of PfEMP1 (Waller *et al*, 1999), an interaction that may be enhanced by phosphorylation by RBC casein kinase II, thereby influencing cytoadherence behaviour (Hora *et al*, 2009), but the effect seen on adhesion after deletion of KAHRP could also be explained by the loss of the knob structures and the concomitant changes in the biophysical display of PfEMP1. The picture generated from these experiments is of a complex interaction involving many components that seem to have a function in the optimal display of PfEMP1, particularly in supporting the role of this molecule in binding to host receptors and underpinning its importance to the parasite. Using gene knockouts a recent study identified several other proteins that play a role in PfEMP1 trafficking, membrane rigidity and cytoadherence through a large screen of ‘transported’ proteins (Maier *et al*, 2008), the function of these newly identified proteins is not well understood but the discovery of several new proteins highlights the complexity of parasite RBC adaptation.

In modifying the host RBC membrane the malaria parasite moves specific proteins from the parasite cytoplasm across the parasite plasma membrane and the parasitophorous vacuole (PV) membrane into the RBC cytoplasm. The details of this process go beyond the scope of this review, however some elegant studies have identified specific sequences in the genes encoding these proteins (known as PEXEL or HT motifs) that identify them for transport across the PV membrane (Hiller *et al*, 2004; Marti *et al*, 2004) and have also described molecular components of the transport pathway, involving Plasmepsin V (Boddey *et al*, 2010; Russo *et al*, 2010). Whilst this part of the process is well defined, the mechanism by which proteins are trafficked to the RBC membrane is less well described. Key components of this latter pathway are the Maurer’s clefts, membranous structures found throughout the erythrocyte cytoplasm although frequently positioned just below the RBC membrane, and visualized in a typical pattern of punctate staining using fluorescence markers (e.g. MAHRP2; Pachlatko *et al*, 2010). Parasite-derived erythrocyte membrane proteins appear to influence the architecture of the Maurer’s clefts as well as using them in transit to the erythrocyte membrane. How these proteins move from the Maurer’s clefts to the erythrocyte membrane is not known but could employ two different mechanisms involving vesicle-like structures or the direct delivery of soluble proteins via the cytoplasm.

Another critical modification to the erythrocyte membrane caused by the parasite is the production of novel transport pathways that can support the development of the parasite during the erythrocytic cycle in a cell with reduced metabolic activity (for review see Martin *et al*, 2009). At around 12 h after the invasion of the RBC the erythrocyte membrane undergoes changes causing increased permeability to a wide range of low molecular weight compounds including amino acids (e.g. methionine and isoleucine), inorganic and organic ions, and nutrients, such as the vitamin pantothenic acid. It is less clear whether the transport pathways induced on invasion are modifications to existing RBC channels or parasite-derived proteins inserted into the erythrocyte membrane. Transporters across the many other membranes within the iRBC are better understood and are of great interest to the malaria research community both in terms of their involvement in resistance to existing antimalarial drugs, but also in their own right as targets for the development of new interventions.

### Pathogenesis: Consequence to the host of membrane and cytoplasmic modifications – decreased deformability

Parasite remodelling of the RBC has consequences for human disease. The human erythrocyte is a highly differentiated cell, specialized to package haemoglobin, carrying oxygen to and carbon dioxide away from tissues. The structure is optimized to withstand high sheer stresses in the arterial circulation and to be highly deformable so that the 8 μm RBC can pass through the 3 μm capillaries in end organs and the 1–2 μm intra-endothelial slits in the spleen (Cooke *et al*, 2001; An & Mohandas, 2008; Maier *et al*, 2009). This considerable feat is achieved through the lack of organelles – giving a low cytoplasmic viscosity, a biconcave structure – giving maximum surface area to volume ratio and through the assembly of the cytoskeletal proteins allowing them to fold into each other and even associate and dissociate – giving the RBC extremely high membrane elasticity (Fung, 1966; Cooke *et al*, 2001; Maier *et al*, 2009). Through modification of the RBC, *P. falciparum* infection interferes with all of these properties, increasing cell rigidity and decreasing deformability (Glenister *et al*, 2002; Shelby *et al*, 2003), contributing to pathogenesis by increasing the tendency to cytoadhere and sequester in microvessels (Cooke *et al*, 2004) and further by impairment of perfusion as rigid iRBCs clog microvessels already reduced in size by sequestration (Dondorp *et al*, 2004). Furthermore, oxidative damage causes decreased deformability of uninfected RBC, which may further impair perfusion and exacerbate anaemia, as uninfected cells fail to pass through the splenic filter and are removed from circulation (Dondorp *et al*, 1999).

As with antigenic variation, the loss of deformability of malaria iRBC was first described in the primate malaria *P. knowlesi* when it was noted that iRBC were less able to pass through polycarbonate sieves and that this effect was more pronounced in more mature forms (Miller *et al*, 1971). As this method did not allow measurement of the effect on individual RBC it was unclear what RBC properties were altered to induce this change. Video rheology (Cranston *et al*, 1984), laser ektacytometry (Dondorp *et al*, 1997) and micropipette techniques (Nash *et al*, 1989) have demonstrated that *P. falciparum* infection leads to decreased deformability through decreased elasticity of the membrane and increased cell size – factors that are increased in more mature parasite stages. More recently, transgenic parasites (Glenister *et al*, 2002, 2009) and microfluidics models (Shelby *et al*, 2003; Antia *et al*, 2008; Herricks *et al*, 2009) have shed further light on the mechanisms of loss of RBC deformability. Gene deletion of parasite proteins has shown that a large proportion of loss of cell deformability is due to the incorporation of the proteins KAHRP and PfEMP3 into the RBC cytoskeleton with KAHRP playing the dominant role, accounting for 51% of the overall increase in rigidity in iRBC (Glenister *et al*, 2002). A screen of gene knockouts has also shown a number of previously undescribed parasite proteins exported to the RBC membrane that contribute to iRBC rigidity (Maier *et al*, 2008). That KAHRP and PfEMP1 – factors assisting in cytoadherence – would account for the majority of iRBC rigidity raises an interesting conundrum. Cytoadherence is thought to protect rigid mature forms from passing through the spleen, yet if gaining the property of cytoadherence causes rigidity this becomes a circular argument. This was further questioned when it was recently demonstrated that *P. vivax*, which also has some capacity to bind to endothelial receptors (Carvalho *et al*, 2010) does not make the RBC more rigid and may in fact make it more deformable (Suwanarusk *et al*, 2004; Handayani *et al*, 2009); implying that increased rigidity is not a necessary consequence of RBC Plasmodial infection or of the capacity to cytoadhere. One possible explanation comes from a recent microfluidics study, which showed that even immature *P. falciparum* rings, which don’t express KAHRP, have a considerably larger volume than uninfected RBC and are likely to be removed in significant numbers by the spleen (Herricks *et al*, 2009). Hence, fundamental differences between these two species may have led them to adapt in different ways to avoid the genetic pressure exerted by the splenic filter, with *P. falciparum* adapting to avoid the spleen by making the iRBC highly cytoadherent and *P. vivax* generating a less cytoadherent but highly deformable iRBC that can successfully pass through the spleen.

#### Effects of the modified iRBC on other intravascular components

Although the parasite does not infect other intravascular cells, through the adapted iRBC *P. falciparum* alters the phenotype of other cells and components within the vessel modifying the intravascular environment, leading to conditions favourable to its survival. We explore some of these effects and how they are implicated in pathogenesis.

#### Microparticles

Microparticles (MP) are small vesicles formed by the budding of membrane from a wide variety of different cells, they are devoid of organelles but maintain the surface receptors of their cell of origin. They have diverse physiological functions but are also involved in the pathogenesis of many conditions, including malaria. Endothelial microparticles, which are pro-coagulant and pro-adhesive have been shown to be increased in Malawian children with CM (Combes *et al*, 2004). More recent studies of malaria patients in Cameroon and Thailand have shown that erythrocytic microparticles are also markedly elevated (Pankoui Mfonkeu *et al*, 2010; Nantakomol *et al*, 2011). In experimental CM, erythrocytic MPs from iRBC, but not uninfected RBC, can induce macrophage activation, as assessed by CD40 upregulation and TNF release (Couper *et al*, 2010). Interestingly, *in vitro*, these iRBC-derived MP triggered higher levels of macrophage activation than intact iRBC themselves. Thereby, erythrocytic MPs qualify as potential effectors of immunomodulation during malarial infection. They are likely to be involved in pathogenesis as well as in the fine-tuning of adaptive immune responses. iRBC can also induce MP from platelets and in an *in vitro* CM model these appear to replicate the effects of platelets on endothelial cells, and particularly to enhance iRBC cytoadhesion (Faille *et al*, 2009a, b).

#### Inflammation and modulation of endothelial cell function by iRBC

Adhesion and sequestration are necessary but not sufficient to explain malaria disease and the importance of inflammation in malaria pathogenesis has been demonstrated in human disease (Grau *et al*, 1989a), as well as in animal (Grau *et al*, 1987) and *in vitro* models of malaria (Wassmer *et al*, 2005). It has become clear that it is unhelpful to see inflammation and iRBC adhesive interactions with host cells as separate entities, as inflammation upregulates endothelial surface receptors and thereby increases adhesion (Ho & White, 1999; Newbold *et al*, 1999). Conversely, iRBC endothelial cytoadherence leads to upregulation of inflammation, although it is debated whether this occurs by iRBC cytoadherence on its own or whether other factors are necessary. In support of an exclusive role of iRBC endothelial interactions, iRBC can induce endothelial cell nuclear factor-κB-regulated inflammatory pathways (Tripathi *et al*, 2009). Such pro-inflammatory effects on endothelial cells may be amplified when iRBC are combined with platelets in co-culture experiments (Wassmer *et al*, 2006) and, more recently, microarray data on iRBC and platelets co-cultured with human brain endothelium indicate that the combination of iRBC and platelets can cause significant pro-inflammatory changes not induced by iRBC alone (Barbier *et al*, 2011). The effect of iRBC is not limited to cytoadherence as haemozoin – produced inside iRBC and released on rupture – can induce NLRP3 inflammasome activation and IL-1β production though the involvement of the Src kinase Lyn and the tyrosine kinase Syk (Shio *et al*, 2009).

iRBC cytoadhesion causes other endothelial cell changes, for which the advantage to the parasite is less clear, but which are nonetheless implicated in pathogenesis. In cerebral endothelial cells iRBC cytoadhesion can cause changes to the blood brain barrier, downregulating cerebral endothelial tight junction formation (Susomboon *et al*, 2006) and enhancing permeability (Yipp *et al*, 2003; Gillrie *et al*, 2007; Tripathi *et al*, 2007). Cytoadhesion can also alter vasomotor function and, at high iRBC doses, has been shown to induce endothelial cell apoptosis (Toure *et al*, 2008). Furthermore recent work has shown that iRBC can trigger the transformation of endothelial cell phenotype; through the transfer of *P. falciparum* antigens onto the endothelial cell surface, thereby possibly converting brain endothelial cells into antigen-presenting cells (Jambou *et al*, 2010) leading to dramatic alterations of trans-endothelial electrical resistance (Jambou *et al*, 2010) and permeability.

**Fig 2:**
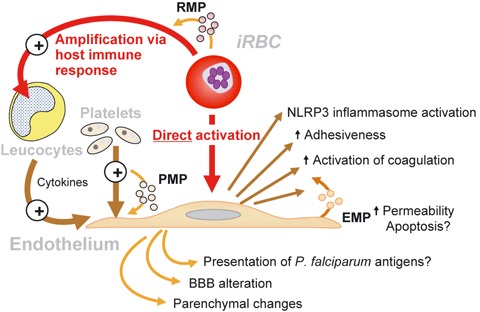
Direct and indirect effects of iRBC on microvascular endothelial cells. In addition to their capacity to directly alter endothelial cells, iRBC and the microparticles they release can trigger potent pro-inflammatory responses, notably by the activation of host leucocytes. These activated cells are responsible for an overproduction of mediators, including cytokines. In turn, cytokine effects can be amplified by platelets. Both platelet-derived and endothelial-derived microparticles also participate in microvascular damage. See text for details. iRBC: infected red blood cell; RMP: red blood cell microparticles; PMP platelet microparticles; EMP endothelial microparticles; BBB, blood-brain barrier.

Nonetheless, the direct effects of iRBCs alone cannot explain the triggering of severe or CM; were this the case then it might be expected that all patients with circulating iRBC would develop CM or at least pan-endothelitis. Consequently, there is a need for ‘something else’ for the triggering of more pathogenic events and an amplification of iRBC-triggered changes by host immune responses is implicated in the shift between uncomplicated and severe malaria (Grau & de Kossodo, 1994) (Fig. 2). Evidence for the role of cytokine overproduction has been demonstrated in experimental (Grau *et al*, 1987, 1988, 1989b) and human CM (Grau *et al*, 1989a; Chizzolini *et al*, 1990; Molyneux *et al*, 1991). The direct role of cell-cell contact in triggering ICAM-1 upregulation in endothelial cells has been demonstrated in the case of leucocytes (Lou *et al*, 1996) and of iRBC (Tripathi *et al*, 2006). Interestingly, some host cells, particularly platelets, can act as effectors of cytokines [reviewed in (Grau & Lou, 1993; Lou *et al*, 1997) and see Pathogenesis: Consequences to the host of parasite surface proteins – cytoadherence and sequestration]. Furthermore, iRBC can directly activate platelets, independently of vascular effects, at least in the experimental murine model (Srivastava *et al*, 2008). Beyond this ‘non-coagulation-related’ platelet involvement, triggering of coagulation by iRBC and its role in severe malaria pathogenesis has been reviewed elsewhere – with the suggestion that the interaction between cytoadherence, inflammation and coagulation is needed to explain CM pathogenesis (van der Heyde *et al*, 2006; Francischetti *et al*, 2007; Moxon *et al*, 2009).

## Conclusion

Years of striving to understand parasite modifications to the RBC have led to a partial grasp of the nature of the remodelled iRBC and the mechanisms used by the parasite to achieve these alterations. These advances have assisted in the understanding of malaria pathogenesis and in the generation of vaccine candidates. From this there has been some notable progress. A picture of malaria pathogenesis involving the combination of different mechanisms is emerging and potential adjunctive therapies aimed at correcting endothelial dysfunction and at the coagulation-inflammation interface hold some promise and further research into these areas is warranted. However, to date there are still no proven effective adjunctive treatments for severe malaria and mortality remains high. On vaccine development there has also been progress gleaned from better understanding of the parasite and its interactions with the host. After many previous failures more recently some vaccine targets have shown significant protection. Nonetheless, although they do prevent disease, they are far from meeting the levels of efficacy considered acceptable for vaccines to other common infections. To assist in closing the gap in our understanding of this complex pathogen, the advent of more powerful sequencing techniques and the ability to knockout parasite genes allow more systematic assessment of the parasite genome and already have revealed that there are genes for many parasite proteins that have not been recognized previously. This technology may be invaluable in the development of effective treatments and vaccines. However the work described in this review highlights how parasite RBC adaptations are frequently only understood when in the context of the host environment with which they interact, either in disease models or in patients. Therefore translating the findings from this new technology into vaccines and treatment will require the consideration of the phenotypic effects of these genes on the iRBC, not just in isolation, but in the context of human infection and disease.
